# Reporting of prognostic markers: current problems and development of guidelines for evidence-based practice in the future

**DOI:** 10.1038/sj.bjc.6600886

**Published:** 2003-04-15

**Authors:** R D Riley, K R Abrams, A J Sutton, P C Lambert, D R Jones, D Heney, S A Burchill

**Affiliations:** 1 Department of Epidemiology and Public Health, University of Leicester, 22-28 Princess Road West, Leicester, LE1 6TP, UK; 2 Department of Medical Education, University of Leicester, University Road, Leicester, UK; 3 Cancer Research UK Clinical Centre, St James's University Hospital, Beckett Street, Leeds, UK

**Keywords:** prognosis, marker, survival analysis, meta-analysis, systematic review, guidelines

## Abstract

Prognostic markers help to stratify patients for treatment by identifying patients with different risks of outcome (e.g. recurrence of disease), and are important tools in the management of cancer and many other diseases. Systematic review and meta-analytical approaches to identifying the most valuable prognostic markers are needed because (sometimes conflicting) evidence relating to markers is often published across a number of studies. To investigate the practicality of this approach, an empirical investigation of a systematic review of tumour markers for neuroblastoma was performed; 260 studies of prognostic markers were identified, which considered 130 different markers.

The reporting of these studies was often inadequate, in terms of both statistical analysis and presentation, and there was considerable heterogeneity for many important clinical/statistical factors. These problems restricted both the extraction of data and the meta-analysis of results from the primary studies, limiting feasibility of the evidence-based approach.

Guidelines for reporting the results of primary prognostic marker studies in cancer, and other diseases, are given in order to facilitate both the interpretation of individual studies and the undertaking of systematic reviews, meta-analysis and, ultimately, evidence-based practice. General availability of full individual patient data is a necessary step forward and would overcome the majority of problems encountered, including poorly reported summary statistics and variability in cutoff level, outcome assessed and adjustment factors used. It would also limit the problem of reporting bias, although publication bias will remain a concern until studies are prospectively registered. Such changes in practice would help important evidence-based reviews to be conducted in order to establish the most appropriate prognostic markers for clinical use, which should ultimately improve patient care.

Prognostic markers (also called prognostic variables or factors) are relevant tools in the management of patients with cancer, and also many other medical conditions, because they help to stratify patients for treatment by identifying different risk groups in order to reduce morbidity and mortality. They include biological, clinical, genetic, histological and pathological features. For example, carcinoembryonic antigen (CEA) is a prognostic marker in colorectal cancer (Eche *et al*, 2001).

An *evidence-based* approach to identifying the most valuable prognostic markers for a given disease is clearly important because it is common for evidence relating to markers to be published across a number of studies, often with conflicting results (Altman, 2001, pp 228–247; Riley *et al*, 2003a). Furthermore, a frequent difficulty in assessing the clinical value of prognostic markers is the relatively small number of patients in primary research studies, sometimes a consequence of disease rarity and limited resources, such that each primary study has low statistical power for detecting any benefits of prognostic staging. The use of systematic review, and in particular meta-analysis, methodology may therefore be important and allow a useful assessment of the prognostic power of markers (Altman, 2001, pp 228–247). A *systematic review* is the preferred means of identifying and combining existing evidence (Egger *et al*, 2001). *Meta-analysis* is the statistical analysis of the review, which seeks to combine all the relevant results found from the literature identified in a quantitative way to produce results more precise than is possible from the individual studies (Sutton *et al*, 2000).

In this paper, we use a recently performed systematic review of prognostic tumour markers studied in neuroblastoma to demonstrate the problems encountered when using this approach, and highlight how they limit evidence-based practice. We then generalise the problems to other areas of oncology, and indeed other disease settings, and ultimately provide specific guidelines for reporting primary prognostic marker studies.

## METHODS

Neuroblastoma is a neuroblastic tumour of the primordial neural crest and is the most common extracranial solid tumour of childhood. The study of prognostic markers for this disease forms an active research area within which a large body of evidence exists. This makes it an appropriate area for an empirical investigation, and as such the problems identified in this study are highly likely to generalise to other disease settings. A brief description of the systematic review strategy adopted is now given.

### Search strategy

The three on-line bibliographic databases Medline, Embase and Cancerlit were chosen as a basis for identifying the relevant literature from 1966 to February 2000. Papers written in a non-English language were excluded. A full description of the search strategy and inclusion/exclusion criteria is provided in Riley *et al* (2003b). One investigator performed the assessment of the papers, with second and third investigators independently checking a sample of them. To be included in the review, a paper had to provide a quantitative result or give tabulated individual patient data evaluating the use of a tumour marker in neuroblastoma from a primary research study of humans. To be classified as relevant to *prognosis*, a paper had to present data, in the form of summary statistics or individual patient data, relating tumour marker levels at a measured point in time to the outcome of patients at the end of a specific follow-up period. Owing to the large number of potential markers, we focused on genetic/biological markers rather than histological markers.

### Data extraction for meta-analysis

From each of the papers included, information was extracted on the tumour markers studied. Meta-analysis of those markers on which eight or more papers provided data was considered. The log_e_(hazard ratio) and its variance were the essential information required from each study, as they provide an important comparative estimate of the risk of death/disease recurrence between two groups of patients. Furthermore, there are several indirect estimation methods available when these statistics are not directly reported (Parmar *et al*, 1998), and the log_e_(hazard ratio) has an approximate normal distribution for large samples, making it particularly amenable to meta-analysis techniques. We make the assumption of proportional hazards throughout this paper.

It was common for a paper to report more than one prognostic result by relating one or more markers to overall survival and/or disease-free survival, and also by providing unadjusted and/or adjusted results (e.g. adjusted for age, stage of disease). Estimates of the log_e_(hazard ratio) and its variance comparing two groups defined by a single marker level were sought from *all* the overall survival and disease-free survival reports. An unadjusted estimate was preferred for each as prior knowledge indicated that adjusted results were likely to be highly inconsistent in the factors for which adjustment was made (Altman, 2001, pp 228–247). An adjusted estimate was sought in the absence of an unadjusted result. Although some markers take only binary values (e.g. chromosome 1p – deletion or no deletion), it was also usual for primary studies to dichotomise continuous variables using a cutoff level in order to categorise patients into high- and low-risk groups.

**Figure 1:**
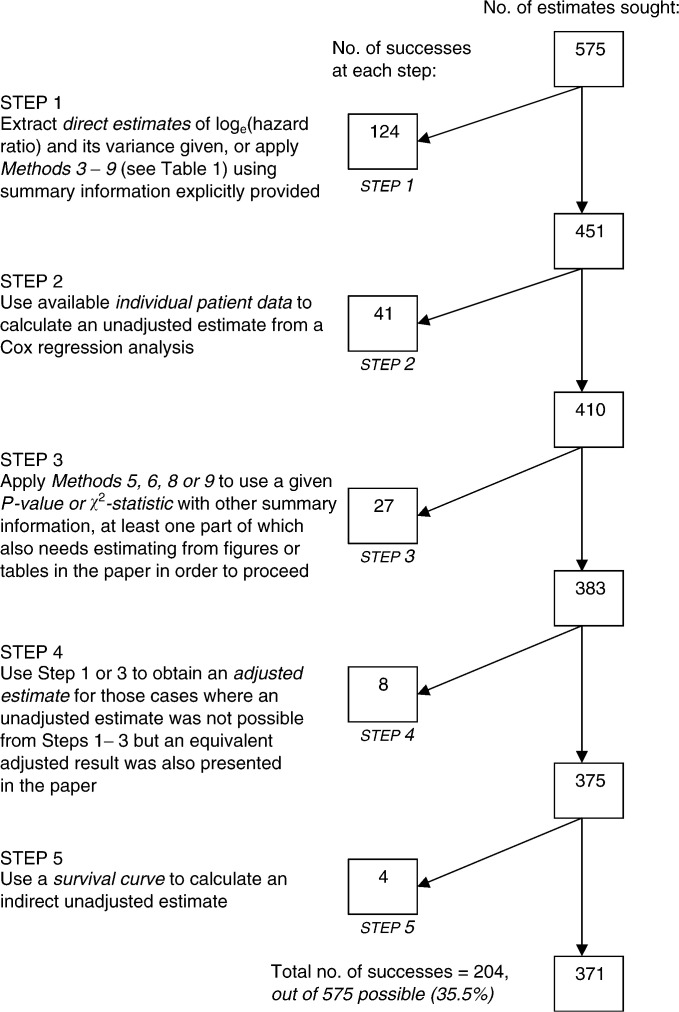
Methods and results at each stage of the sequential process used to obtain a single direct or indirect estimate of the log_e_(hazard ratio) and its variance for each of the reports where one of the 13 tumour markers was related to overall or disease-free survival by summary statistics or individual patient data across the literature. Five steps were used, with unadjusted estimates sought primarily in each *unless* only an adjusted result was available or otherwise stated.

A five-step sequential process (Figure 1

) using 10 different direct and indirect methods (Table 1

**Table 1:** Description of the methods used to obtain estimates of the log_e_(hazard ratio) and its variance

**Method**	**Summary statistics or data required**	**Step 1**	**Step 2**	**Step 3**	**Step 4**	**Step 5**	**Total**
1	HR or log_e_(HR) and V	3	—	—	0	—	3
2	Individual patient data	—	41	—	—	—	41
3	log_e_(HR) and CI	0	—	—	1	—	1
4	HR and CI	30	—	—	3	—	33
5	log_e_(HR) and *P*-value	2	—	0	2	—	4
6	HR and *P*-value	10	—	2	2	—	14
7	HR, group numbers and total events	2	—	—	0	—	2
8	*χ*^2^-statistic, group numbers and total events	10	—	4	0	—	14
9	*P*-value, group numbers and total events	67	—	21	0	—	88
10	Survival curve	—	—	—	—	4	4
							
	Total	124	41	27	8	4	204

The summary statistics required for each method are shown together with the number of times each method was successfully used in Steps 1–5 of the extraction process. Methods 3–10 were used in order of preference shown. HR=hazard ratio; V=variance of the log_e_(HR); CI=confidence interval.

), based on the approach of Parmar *et al* (1998), was used in an attempt to obtain the log_e_(hazard ratio) and its variance. Studies with samples smaller than 25 were not included in Steps 2–5 because they were not considered large enough to justify estimation methods. A more detailed version of Figure 1 and a more in-depth description of the extraction procedure are provided in Riley *et al* (2003b).

## RESULTS

### Literature search results

A total of 3415 papers were identified from the literature search. After assessment, 260 papers were classified as ‘relevant’ to prognosis, and these studied a total of 130 different tumour markers for risk stratification of patients (for references see Riley *et al*, 2003b).

### Data extraction of prognostic marker results

The 13 most commonly studied prognostic markers were each selected for an in-depth study to establish their individual value as a prognostic tool (Table 2

**Table 2:** Names of the 13 markers grouped by tumour marker class, with the number of prognosis papers identified for each, the number of reports when it was related to either overall or disease-free survival by summary statistics or individual patient data, and the number of successful estimates made of the log_e_(hazard ratio) and variance; evidence of heterogeneity is shown for outcome, cutoff levels, age, stage and adjustment factors

**Marker class**	**Marker name**	**Papers^a^**	**OS and DFS reports^a^**	**Total successful estimates (*ψ*)^b^**	**OS/DFS successes^c^**	**(i) Different cutoff groups^d^**	**(ii) Different stage groups^d^**	**(iii) Different age groups^d^**	**U/A^e^**	**Different sets of adjustment factors^f^**
DNA or chromosome abnormalities	*MYC-N*	151	194	94	48/46	9	9	4	77/17	16
	DNA index	44	62	19	11/8	8	3	3	18/1	1
	Chromosome 1p	40	49	20	11/9	1	5	2	18/2	2
										
Urinary catecholamines	VMA	36	40	4	3/1	4	3	2	4/0	—
	HVA	26	29	2	2/0	2	2	1	2/0	—
	VMA:HVA	20	28	5	2/3	3	4	2	5/0	—
	Dopamine	10	11	2	1/1	2	2	1	2/0	—
										
Biological markers	CD44	8	8	3	0/3	1	1	2	3/0	-
	TrkA	16	21	11	4/7	7	1	1	9/2	2
	NSE	28	39	9	4/5	6	3	1	8/1	1
	LDH	26	30	12	5/7	5	4	1	8/4	4
	Ferritin	33	41	7	3/4	5	4	2	6/1	1
	MDR	16	30	16	9/7	8	3	3	13/3	2

VMA=vanillylmandelic acid; HVA=homovanillic acid; NSE=neuron-specific enolase; MDR=multi-drug resistance protein; LDH=lactate dehydrogenase.

a Number of papers reporting prognostic marker results or IPD, with the overall no. of OS and DFS reports within them. There were 211 papers overall which reported overall survival (OS) and/or disease-free survival (DFS) results or individual patient data (IPD) for one or more of the markers.

b Number of OS and DFS reports for which successful estimateswere extracted.

c Number of total successful estimates (*ψ*) by OSand DFS.

d The total successful estimates (*ψ*) could also be grouped by those (i) using the same cutoff level (cutoff includes a group for when it was ‘unknown’ (for an example see Table 3)); (ii) relating to patients with the same stages of disease (stages of disease groups were ‘unknown’ and combinations of stages 1, 2, 3, 4, 4s); and (iii) relating to patients with the same age range(age groups were ‘all ages’, ‘<1 year’, ‘>1 year’ and ‘unknown’). Columns 7–9 show the number of different subgroups in each case.

e Number of total successful estimates (*ψ*) that were unadjusted (U) and adjusted (A) (unadjusted estimates were preferred where possible).

f Number of successful adjusted estimates (A) that related todifferent sets of adjustment factors.

). Expression of CD44 gene was studied in eight papers and the other 12 markers were studied in 10 or more prognosis papers (Table 2). This involved 211 (81.2%) of all the prognosis papers. Within these there were *575 reports* of prognostic power assessment where levels of any of these 13 tumour markers were related to overall survival or disease-free survival by summary statistics or IPD.

Only 204 (35.5%) estimates of both the log_e_(hazard ratio) and its variance could be calculated from Steps 1–5 using Methods 1–10 (Figure 1, Table 1). In particular, the log_e_(hazard ratio) and its variance were both *directly* provided on only three occasions in the 575 reports (0.005%) (Table 1, Method 1), and all were from a single paper (Berthold *et al*, 1997). Fortunately, individual patient data were frequently presented within this literature, and from this a further 41 direct estimates were made (Step 2, Method 2). The remaining 160 successful estimates were obtained using Methods 3–10 (Table 1), the most frequently required of which used a *P*-value/*χ*^2^-statistic in combination with group numbers and total number of events, that is, deaths/recurrences of disease (102 times) (Methods 8 and 9).

### Problems limiting meta-analysis

#### Poor reporting of primary studies

**Figure 2:**
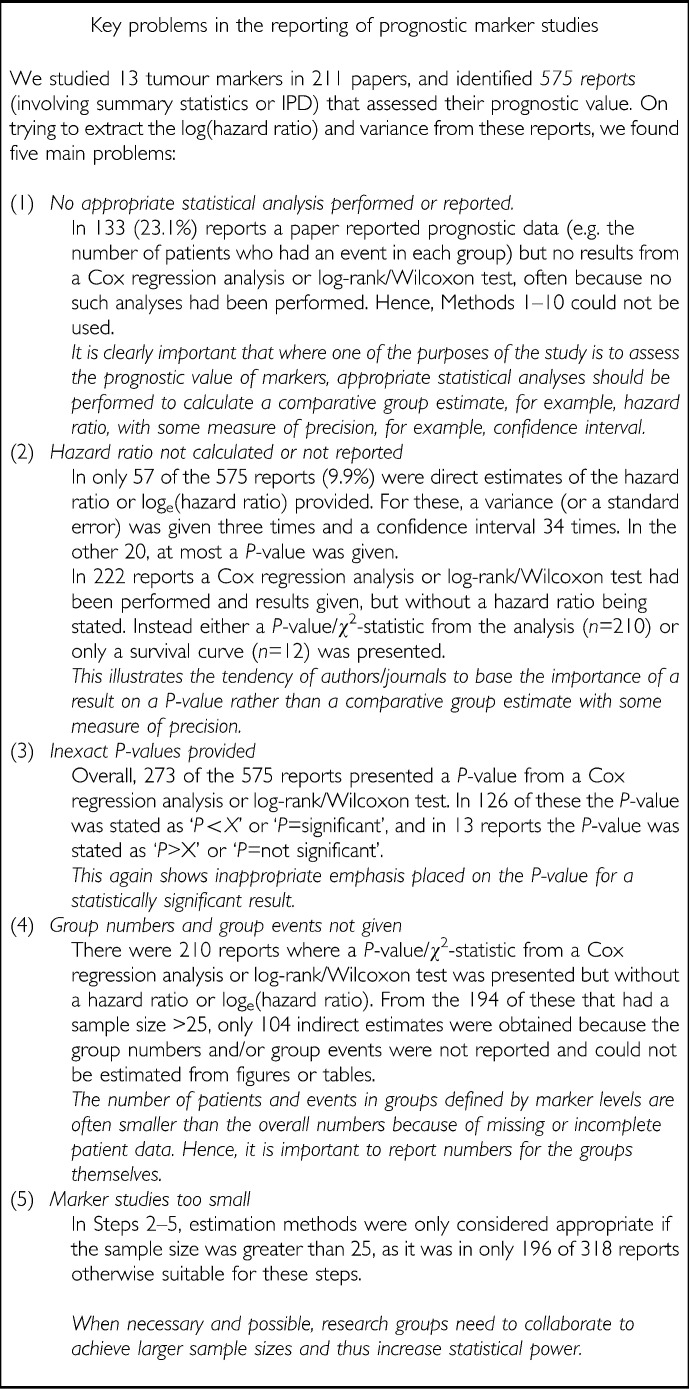
Description of the key reporting problems that prevented estimation of the log_e_(hazard ratio) and its variance in 371 (64.5%) of the reports

Primary studies of prognostic tumour markers are clearly essential and we observed many important results across the literature that have implications for clinical practice. However, the general standard of reporting primary studies was inadequate, and it was disappointing that we only managed to obtain 35.5% of the estimates required despite the intensive, time-consuming extraction procedure (Figure 1). This hindered the use and interpretation of meta-analysis because we could not incorporate the majority of results reported in the literature and consequently introduced a strong potential for bias. Among the 371 reports that did not enable estimates to be made, there were five common reporting problems, most of which can be simply addressed (Figure 2

. Encouragingly, there was some evidence that the reporting of prognostic markers has improved over the last 10 years because all the papers that did provide a hazard ratio or log_e_(hazard ratio) were published after 1990. However, these papers still only represented approximately 17% of the total literature identified over this period (i.e. only 26 out of 157 papers published after 1990 reported a hazard ratio).

#### Heterogeneity of clinical and statistical factors

The synthesis of our estimates was also restricted by the large variability in both clinical and statistical factors. For each estimate of the log_e_(hazard ratio) and its variance obtained, the cutoff level used to dichotomise the continuous markers, stage of disease, age of patients and outcome (overall or disease-free survival) were recorded, and also whether the estimate was unadjusted or adjusted and, if so, what adjustment factors were used. There was great diversity in these features (Table 2). For example, for the marker *MYC-N* there were 94 estimates of the log_e_(hazard ratio) and variance obtained but these involved nine different cutoff points, nine different stage groups, four different age groups, 77 unadjusted/17 adjusted estimates and two different outcomes (Table 3

**Table 3:** Heterogeneity in the 94 estimates of the log_e_(hazard ratio) and its variance obtained for marker *MYC-N*

	***n***
*Outcome*	
* *DFS	46
* *OS	48
	
*Result type*	
* *Unadjusted	77
* *Adjusted	17
	
*Stage groups*	
* *All	68
* *1	2
* *3	2
* *4	4
* *1, 2, 3	3
* *1, 2, 3, 4	5
* *2, 3, 4, 4S	2
* *3, 4	3
* *Unknown	5
	
*Cutoff point*	
* *1 copy	23
* *2 copies	1
* *3 copies	17
* *4 copies	5
* *5 copies	2
* *10 copies	18
* *Mean gene expression	2
* *Positive *vs* negative protein (or staining *vs* no staining)	9
* *Unknown	17
	
*Age groups*	
* *All	78
* *<1 year	2
* *>1 year	5
* *Unknown	9

OS=overall survival; DFS=disease-free survival.

). Furthermore, of the 17 estimates that were adjusted for other prognostic markers or clinical features (using a Cox regression model) only two were adjusted for exactly the same set of factors, and these were from the same article (Maris *et al*, 2000).

This inconsistent and variable reporting was reflected equally in the estimates obtained for the other 12 markers (Table 2). The type of treatment of patients and the method of measuring the markers were not recorded, but both would have added further heterogeneity to that observed.

#### Publication bias and reporting bias

The common problem of publication bias, and other reporting biases, may still affect our data extraction; some results that do not generate formal statistically significant or clinically valuable findings may not have been published, because of a reluctance of journals to report or of researchers to present negative findings. Such problems severely limit the conclusions that can be drawn from meta-analyses because not all the available evidence can be included, and therefore the pooled results are likely to be biased. We investigated the estimates obtained for *MYC-N* and indeed there did appear to be evidence of publication bias, with a number of studies with smaller hazard ratios considered to be missing (Riley *et al*, 2003). This problem is likely to be closely related to the problem of small sample sizes in some primary studies (Figure 2, key problem 5).

### Should we proceed with meta-analysis?

The poor reporting, potential for publication bias and, in particular, the large heterogeneity across studies meant it was practically impossible to perform reliable meta-analyses that would determine the clinical importance of each marker studied. Even the analysis of subgroups of estimates was not considered realistic because it was virtually impossible to obtain subgroups that reflected patients with similar features. For example, for marker *MYC-N* there were 48 overall survival estimates obtained, of which 41 were unadjusted, and 30 related to ‘all’ stages and ‘all’ ages. Furthermore, only eight of these 30 estimates related to the most commonly used cutoff level of ‘1 copy number’, and there is then the additional problem of heterogeneity for treatment used and method of measuring the marker, not to mention the potential impact of publication/reporting bias. The subgroup numbers were even smaller for the other, less-studied markers; for example, lactate dehydrogenase had only two unadjusted overall survival estimates relating to the most common cutoff level (1500 U l^−1^) and patients of ‘all’ ages and ‘all’ stages.

The only possible benefit of meta-analysis using the estimates that we extracted is to highlight the results of previous studies and help prioritise which markers should be studied in the future. We take such an approach elsewhere (Riley *et al*, 2003b), but for the purposes of this feasibility study it is clear that no firm clinical policy decisions can be made from our evidence-based review.

## DISCUSSION

### Appraisal of the systematic review and data extraction

During the systematic review, we evaluated 3415 papers overall and identified 260 with results from studies assessing the prognostic power of tumour markers. This will have identified the majority of the English-language literature, but inevitably some papers will have been excluded unintentionally. However, it seems plausible that the reporting in such papers, and equally non-English papers, would be equally poor and heterogeneous.

We used the indirect methods suggested by Parmar *et al* (1998) to increase the number of occasions an estimate of the log_e_(hazard ratio) and its variance could be obtained. However, the estimates they provide are only approximate and simply make the best possible use of the results presented. Questions still exist about how best to combine indirect estimates with direct estimates. For this reason, we did not use other indirect methods. For example, given further assumptions, we could have used estimates of the proportion surviving to 2, 3, 5 or 10 years to obtain estimates of the log_e_(hazard ratio) and its variance (Vale *et al*, 2002). However, the papers were equally inadequate at presenting these survival statistics. For example, in the 26 prognosis papers for the serum marker lactate dehydrogenase, only 12 gave actuarial estimates of the proportion surviving, and only six of these also gave a confidence interval or standard error. They were also heterogeneous–five estimates were for overall survival, six were for disease-free survival and one was unspecified; estimates were made at 2, 3, 4 or 5 years. Further, very few reported numbers at risk explicitly, as required for reliable estimation.

### Generalisations to other prognostic markers

Although these reporting problems were observed for tumour markers within the neuroblastoma literature, they have also limited reviews in other paediatric cancers (Riley *et al*, 2003a), and it seems plausible that the reporting will be equally poor for prognostic markers in other areas of oncology, and indeed other disease areas. Altman (2001, pp 228–247) discusses the potential problems involved in systematic reviews of prognostic markers, in particular that of poor and heterogeneous reporting of primary studies. Cutoff points are frequently used to dichotomise continuous markers and define groups, while different outcomes, adjustment factors and groups of patients are common features across prognostic studies. Inadequate reporting and presentation of survival data has been shown to be a concern in the cancer literature (Altman *et al*, 1995).

Reliable and clinically useful meta-analyses of observational and nonrandomised studies, such as the majority of prognostic marker studies, are generally difficult to perform (Fleiss and Gross, 1991). Other recent systematic reviews of prognostic markers have encountered similar problems to the ones we identified. Parker *et al* (2001) performed a systematic review in prostate cancer to establish whether age is a prognostic marker, but the incomplete and heterogeneous nature of the reports prohibited any quantitative overview. Similarly, a systematic review of prognostic laboratory variables in patients with unresected colorectal liver metastases was limited by the heterogeneity and poor quality of individual studies (Friedburg *et al*, 2001). Zandbergen *et al* (2001) performed a systematic review of biochemical markers of brain damage for identifying poor outcome in anoxic-ischaemic coma, but conclusions were limited by small sample sizes and different cutoffs and/or laboratory techniques.

Meta-analyses of prognostic markers have been facilitated when individual patient data were available (Look *et al*, 2002), in particular to determine a consistent cutoff level (Sakamoto *et al*, 1996). For those investigators currently interested in performing a quantitative review of prognostic markers, we recommend that they consider asking authors for individual patient data and/or the extra information they require, such as the log_e_(hazard ratio) and its variance, as this approach is likely to be the most productive.

### Towards guidelines for improved reporting of prognostic markers

**Figure 3:**
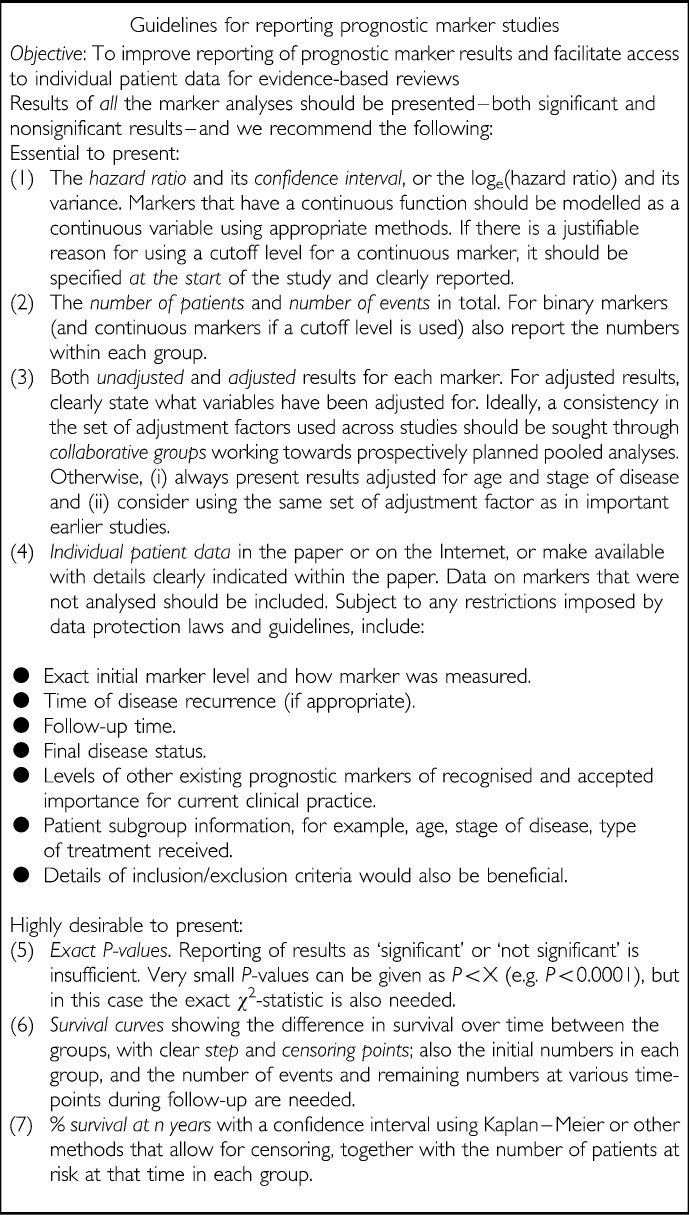
Guidelines on how to report primary prognostic marker studies in order to improve current reporting standards and allow clinically useful evidence-based reviews to be made

It is clearly important that the quality of primary studies, and the reporting of their results improve if clear conclusions and policy recommendations are to be formed about prognostic markers. Altman and Lyman (1998) have proposed important guidelines for both conducting and evaluating prognostic marker studies, including the need for prospective registration of studies. Alongside these, we have developed simple guidelines on how to report results to facilitate both interpretation of individual studies and the undertaking of systematic reviews, meta-analysis and, ultimately, evidence-based practice (Figure 3

. Collaboration of research groups is required to promote such practice and achieve both the consistency and standards required. Ideally, *both* summary data and individual patient data should be reported according to our guidelines. It is important that *time to event* is incorporated within prognostic marker analyses, and thus the hazard ratio is preferred to other measures of relative risk such as the odds ratio, which relates to a fixed time-point and ignores censoring. However, in addition authors may wish to present the more familiar actuarial % survival at *n* years preferably with a confidence interval and the number of patients at risk at that time in each group.

#### Benefits of individual patient data

Although improved reporting of summary statistics is very important, the availability of individual patient data is the most viable way forward in order to produce valid and clinically useful evidence-based reviews of prognostic markers. Subject to any restrictions imposed by data protection laws and guidelines, presentation or availability of full individual patient data using our guidelines would overcome variability in cutoff level, type of estimate (unadjusted or adjusted), outcome assessed (overall or disease-free survival) and adjustment factors; the study of markers in subgroups of patients (e.g. different ages, treatments) would also be easier. It would also eliminate the problem of extracting estimates when inexact *P*-values are presented, and would remove the need for arbitrary extraction decisions when an individual study presents a marker's results for a range of cutoff values. Furthermore, if levels of all the prognostic markers measured (even those producing nonsignificant results) are provided, then the problem of reporting bias would be reduced. However, publication bias might still be a concern if some studies are not published and do not make IPD available; prospective registration of studies is therefore also important to counteract this.

Individual patient data would also enable direct estimates of the hazard ratio, and other statistics of interest, when data were available but not used, analysed or presented properly in the primary study. A total of 41 (20%) of the 204 estimates that we obtained in the neuroblastoma review were direct estimates calculated from individual patient data that would not have otherwise been possible. It is clearly important to include predominately direct estimates in any quantitative synthesis. In fact, the potential for substantial differences in meta-analysis of survival data when using results provided within the literature instead of individual patient data has recently been shown in the head and neck cancer literature (Duchateau *et al*, 2001). Individual patient data would also allow model assumptions, for example proportional hazards, to be checked as necessary, and enable the baseline survival function to be estimated.

Presentation or availability of individual patient data would permit more appropriate meta-analyses (Stewart and Parmar, 1993), and would further facilitate the identification of different publications whose results relate to the same or overlapping set of patients. It would also allow an evaluation of combinations of markers, which may produce more specific and accurate prognostic assessments. If it is not appropriate or feasible to provide individual patient data within a paper itself, then there is the opportunity to publish on the Internet (Hutchon, 2001). Of course, even making individual patient data available on the web is not without its problems, with the nonpermanency of individual web-pages, and so perhaps a central repository to collate and manage individual patient data is needed within each disease area. The United Kingdom Children's Cancer Study Group have already initiated this type of approach within paediatric oncology (Mott *et al*, 1997). Authors may also wish to state in their paper that the IPD is available upon request (with contact details indicated) for those requiring it for evidence-based reviews.

We acknowledge that there are additional issues that arise when conducting individual patient data reviews (Stewart and Clarke, 1995), especially cost and time, but these have to be weighed against the substantial problems we encountered. Of course, even when prioritising the IPD approach, the meta-analyst will in practice end up with a mixture of estimates obtained from IPD and estimates obtained from summary statistics; hence, meta-analysis methods that take these different sources into account are needed.

#### Cutoff levels

The use of different cutoffs makes synthesis of results particularly difficult. Of added concern is the possibility that the choice of cutoff level in a report may be specifically chosen to optimise the difference between the groups and produce a result with the maximum statistical or clinical significance possible (Altman *et al*, 1994; Altman and Lyman, 1998). If there is good clinical reason to use a cutoff level, then it should be specified at the start of a study and clearly reported within the results (Figure 3). However, Altman (2001) suggests that continuous markers should not be dichotomised because, among other reasons, this approach discards potentially important quantitative information and considerably reduces the power to detect a real association between the marker and outcome. Hence, we encourage researchers to analyse and report results (e.g. hazard ratio) of continuous markers on their original continuous scale. Importantly, availability of individual patient data including *exact* marker levels would allow data to be reanalysed where cutoff levels were not consistent, and also where continuous marker results were desired but results using a cutoff level were given (or *vice versa*) (Figure 3). Indeed, the most appropriate analysis of continuous prognostic markers may require nonlinear modelling techniques, as highlighted by Sauerbrei *et al* (1999); consultancy with statisticians or others experienced with such techniques is recommended in this situation.

#### Adjustment factors

It is clear that once important prognostic markers have been identified, they need to be evaluated against, and also used in combination with, other known clinically useful prognostic factors, such as clinical characteristics (e.g. age, stage of disease) or indeed other marker levels. Prognostic marker results that are adjusted for other known prognostic factors will have the greatest implications for clinical practice, and subsequently meta-analyses of adjusted results are the necessity. However, if authors are inconsistent in the sets of adjustment factors they use, it becomes very difficult and impractical to pool results across studies and make a proper evaluation of markers over and above other factors. For the 17 adjusted *MYC-N* estimates, there were 16 different sets of adjustment factors, each containing one or more of age, stage of disease, Shimada index, lactate dehydrogenase and eight other prognostic markers. Individual study estimates of risk (e.g. hazard ratio) can be influenced by which adjustment factors are used (Rushton and Jones, 1992), and so there may be an additional reporting bias concern if researchers specifically only report those adjusted estimates with the most statistically significant result.

We recommend that research groups collaborate and identify the most commonly used prognostic tools in current practice, so that adjusted results of new prognostic marker studies can use consistent sets of adjustment factors. These identified prognostic tools should also be presented within the available individual patient data alongside the new markers being studied (Figure 2). This would allow adjusted results to be calculated independently across studies, using consistent sets of adjustment factors. Prognostic indexes could also be calculated across studies and evaluated in a meta-analysis if desired. Lambert *et al* (2002) have shown that individual patient data are generally required when investigating patient characteristics as effect modifiers in a meta-analysis, and, for prognostic markers, our study shows that the most valid and clinically useful meta-analysis results will only be obtained from an individual patient data approach.

## CONCLUSION

Prognostic markers are important tools in the management of patients with cancer and many other diseases, and as such primary studies of prognostic markers are essential. However, the design and evaluation of such studies can be greatly improved (Altman and Lyman, 1998). Furthermore, we have shown that a change in how prognostic marker studies are reported is needed to provide more effective and meaningful results, and also allow important *evidence-based* reviews to be conducted. To facilitate such improved reporting, we have attempted to compile guidelines regarding how summary statistics and individual patient data should be presented. In particular, the availability of full individual patient data, including all markers considered, is the most viable way forward to produce valid and clinically useful evidence-based reviews and meta-analyses. Individual patient data would limit the large problems of poor and heterogeneous reporting that we observed, and also reduce the potential impact of reporting bias. Prospective registration of studies alongside the availability of IPD would also help restrict the potential for publication bias. These guidelines all point to researchers working together towards planned pooled analyses, currently a particularly important concept for epidemiological research (Blettner *et al*, 1999).

Research groups within each disease area should be encouraged to collaborate and facilitate these changes in practice; for example, by defining a clear set of important adjustment factors and by initiating central repositories to collate and manage individual patient data. This move towards a more evidence-based approach to the study and reporting of prognostic markers will help properly establish the most appropriate individual, and potential combinations of markers to be used in clinical practice, and should thereby improve patient care.
